# A retrospective case–control study of a cluster of surgical site infections after coronary artery bypass grafting at a tertiary medical center

**DOI:** 10.1017/ash.2025.164

**Published:** 2025-06-11

**Authors:** Alaina S. Ritter, Vidya Kollu, Amanda Aspilcueta, Jennifer D. Connolly, Eddie Manning, Lennox Archibald

**Affiliations:** 1 Division of Infectious Diseases and Global Medicine, College of Medicine, University of Florida, Gainesville, USA; 2 Division of Infectious Diseases, North Florida South Georgia VA Medical Center, Gainesville, FL, USA; 3 Infectious Disease, HCA North Florida Hospital, Gainesville, FL, USA; 4 Infection Control Division, Malcom Randall Veterans Affairs Medical Center, Gainesville, USA (Retired); 5 College of Nursing, North Florida South Georgia VA Medical Center, Gainesville, FL, USA; 6 Cardiothoracic Surgery Section, North Florida South Georgia VA Medical Center, Gainesville, FL, USA

## Abstract

**Objective:**

To investigate a cluster of surgical site infections (SSIs) in patients who underwent coronary artery bypass graft (CABG) procedures, identify risk factors for infection, and implement measures to prevent new cases.

**Design:**

The investigation comprised a retrospective case–control study and an observational review of infection control practices between the fall of 2018 and 2019 (study period).

**Setting:**

Tertiary care medical center in Florida, USA.

**Patients:**

Patients who acquired an SSI following CABG during the study period were defined as case-patients. Control-patients were randomly selected patients who did not acquire a post-CABG SSI.

**Methods:**

We recorded clinical and epidemiologic details on a standardized form and analyzed data with SAS statistical software. Odds ratios and 95% confidence intervals were calculated.

**Results:**

Seven patients met the case definition and 21 control-patients were identified. While multiple variables were significant on univariate analysis, after controlling for confounding using multivariate analysis/logistic regression, only lower age (*P* < 0.0001) and meeting the requirements for appropriate perioperative temperature management (SCIP measure 10) (*P* = 0.01) were identified as independent risk factors for SSI. Per observational review, measures to reduce operating room traffic and limit door opening/closing were implemented and wound vacuum-assisted closure (VAC) use was phased out. Our institutional SSI rate returned to baseline and no additional clusters were seen in the following three years.

**Conclusions:**

Multiple potential risk factors exist for SSI after coronary artery bypass grafting. At our institution, minimizing operating room traffic and reducing wound VAC use may have successfully addressed these healthcare-associated infections.

## Introduction

Coronary artery bypass grafting (CABG) is the most common cardiac surgical procedure performed in the United States.^1^ Surgical site infection (SSI) following CABG is a significant cause of morbidity and mortality.^2–4^ Mitigating strategies include adherence to mandatory preoperative preventive requirements, including those established by the Surgical Care Improvement Project (SCIP) along with the Joint Commission. Despite these measures, approximately 1 to 4% of patients acquire infections after undergoing CABG.^2,3,5–7^ The most common causative organisms in sternal wound infections are *Staphylococcus* species and gram-negative bacilli.^8^ Deep SSIs, including mediastinitis, are associated with increased mortality rates.^9^ Previous investigations of risk factors for SSI after CABG have yielded variable results.^2,10–12^

Between the fall of 2018 and 2019, an increase in the occurrence of SSIs in patients who underwent a CABG procedure at our facility was noted despite standard SSI prevention measures, which included preoperative chlorhexidine skin preparation and intranasal povidone-iodine for all preoperative patients. Based on prior hospital surveillance data of SSI rates, these occurrences qualified a new cluster of SSIs and an investigation was initiated. The objectives of the investigation were to identify risk factors for SSI as well as implement measures to prevent additional cases.

## Methods

This study was approved by our institutional research compliance office. The need for patient consent was waived given that the study was determined not to meet criteria for human subjects research through use of the VA Electronic Determination Aid (VAEDA).

### Case definition and case ascertainment

A case was defined as any adult patient who acquired an SSI (superficial, deep, and organ/space) within 90 days of CABG surgery at this tertiary care medical center in Florida, USA during a 13-month period between the fall of 2018 and 2019 (study period). Case-patients were ascertained by review of the hospital’s infection control surveillance data. We compared the rates of all post-CABG SSIs during the pre- and post-cluster periods to identify the study period.

### Case–control study

To identify patients at risk of acquiring an SSI, we compared the case-patient population with a randomly selected group of control-patients for a case to control ratio of 1:3 to ensure adequate power. The control population was defined as any adult patient who underwent a CABG surgical procedure during the study period but did not acquire an SSI. SSIs were classified as superficial, deep, or organ/space infections involving the primary CABG surgical site based on National Healthcare Safety Network (NHSN) criteria.^13,14^ Variables analyzed included demographics, past medical history and co-morbidities, operating room procedures and timing, specific healthcare personnel involved in a patient’s care during surgery, vascular and airway access, surgical complications, duration of surgery, duration of medical device use, adherence to the SCIP measures and other perioperative standards of care (including surgical skin preparation, appropriate hair removal with clippers in the peri-operative area, appropriate timing and dosing/re-dosing of peri-operative antibiotics, and peri-operative maintenance of normothermia and normoglycemia), signs and symptoms of infection, exposure to and duration of cardiac bypass, enteral feeding, mechanical ventilation, urinary catheterization, types and duration of intravascular access including postsurgical intracardiac lines and devices, types and duration of intravenous antimicrobial therapy, receipt of blood products, and clinical outcomes.

### Procedural review

Upon identification of the cluster in the second month of the study period, infection preventionists immediately conducted audits using standardized checklists to assess adherence to infection control practices and procedures in the operating rooms, hand hygiene, and environmental services cleaning protocols, including the cleaning of OR equipment. Of note, environmental services was adequately staffed and no changes were made regarding the type of cleaning products used or the frequency/location of cleaning. Any lapses found during audits were conveyed immediately to involved staff, and the OR manager and nursing cardiothoracic surgery leader subsequently monitored and enforced ongoing compliance.

Given ongoing cases despite the above measures, infection prevention personnel subsequently sought additional opinions and feedback from OR staff. These informal conversations occurred in group and individual settings. Staff raised concerns about operating room traffic during CABG procedures and infection preventionists directly observed the perfusion team removing their machinery when cardiac bypass was complete, resulting in prolonged door opening. As a result, policy was changed so that all equipment was left in place until the chest was closed. This change was implemented in the 11^th^ month of the study period.

One surgeon also self-reported increased operation durations in part due to teaching activities and trainee involvement. As a result, additional limitations were placed on the time allotted to trainees during procedures. The cardiothoracic surgery team also reported that prior to the study period, only infected wounds healing by secondary intention received a vacuum-assisted closure device (wound VAC). Around the time of the study period, however, the VAC manufacturer provided new data supporting use in patients at higher risk of infection or dehiscence, leading to expanded use at our institution. In the 13^th^ month of the study period, VAC use was phased out in favor of alternative methods, including surgical glue and VAC systems compatible with surgical glue dressings.

**Figure 1.**
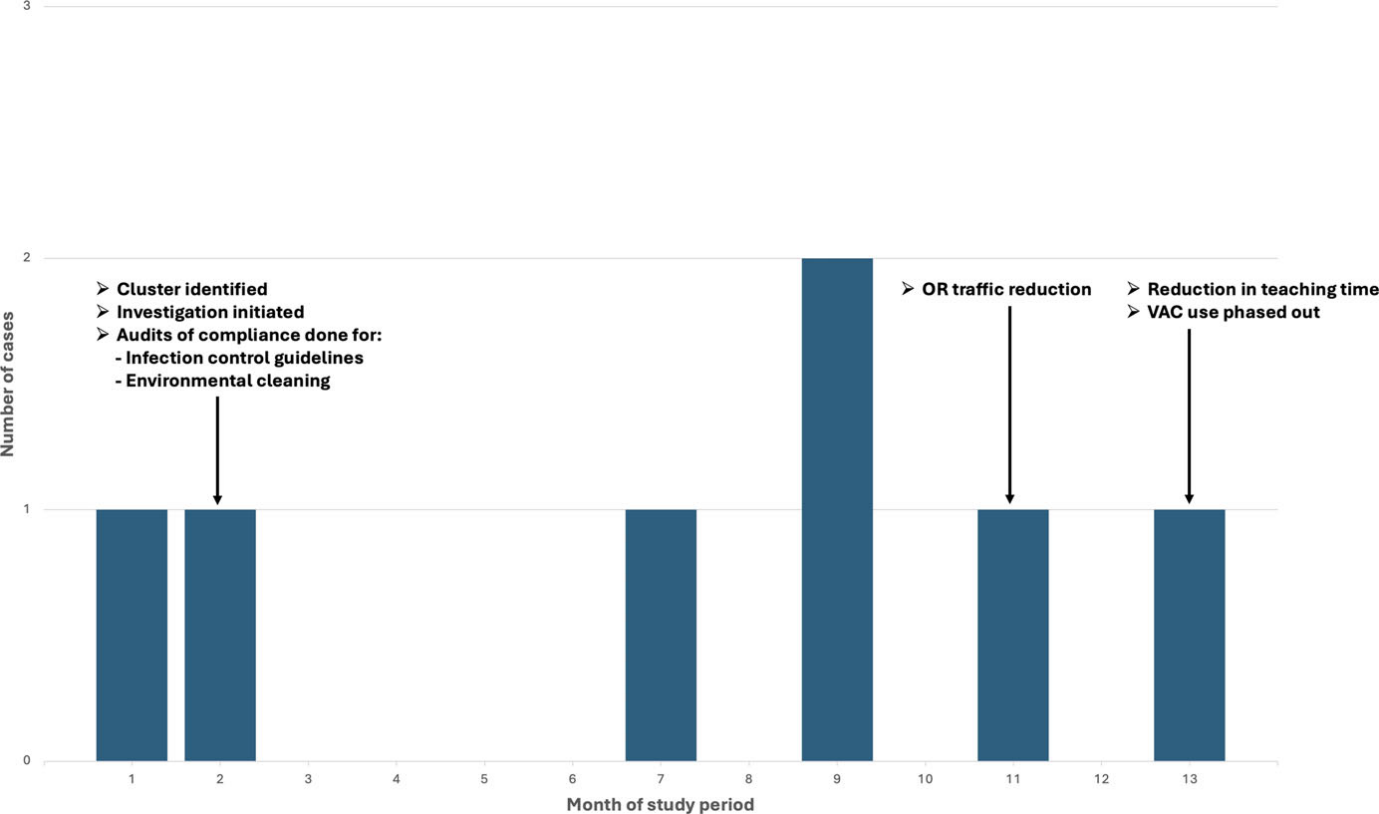
Distribution of cases during the study period including the timing of associated interventions.

See Figure 1 for a graphical representation of the case distribution within the cluster as well as timing of the above interventions.

### Statistical analysis

All data were recorded on standardized forms, entered electronically, and analyzed using SAS statistical software Version 9.4 (SAS Institute Inc, Cary, North Carolina). Categorical variables were compared using the likelihood ratio test or, where appropriate, Fisher’s Exact test. Medians of continuous variables were compared using the Wilcoxon two-sample test. Odds ratios (OR) and 95% confidence intervals (C_95_) were calculated. *P* < 0.05 was considered statistically significant. We performed multivariate analyses and logistic regression to control for confounding variables and effect modification and to identify independent risk factors among those found to be statistically significant on univariate analysis.

## Results

### Case ascertainment and characteristics

During the study period, 118 total CABG surgeries were performed by two surgeons (58 by Surgeon A and 60 by Surgeon B). Seven adult patients met the case definition. Twenty-one control patients were randomly selected. All seven patients who met the case definition were male; six were Caucasian and one was Black. All of the control patients were Caucasian males. The median age of case-patients was 56 (range: 43–67) years; median body mass index (BMI) was 36 (range: 29–39) kg/m^2^. Of the seven patients meeting the case definition, two had superficial SSIs while five had deep SSIs per NHSN classification of surgical wound infections.^13,14^ Two of the five patients with deep SSIs also had mediastinitis. Four patients had surgical cultures positive for methicillin-susceptible *Staphylococcus aureus* (MSSA), one patient had a culture positive for methicillin-resistant *Staphylococcus aureus* (MRSA), one patient had a culture positive for *Escherichia coli*, and surgical cultures were not obtained for one patient. Four patients had secondary bloodstream infections. Of note, molecular analysis was not performed on the MSSA isolates given that the associated surgeries were performed in months 1, 2, 8, and 9 of the study period and a common source of infection was felt to be unlikely. There were no patient deaths attributable to SSI, although one patient died from COVID-19 pneumonia over two years after his CABG surgical procedure.

### Univariate and multivariate analysis of risk factors for SSIs after CABG

Case- and control-patients were similar regarding the presence of underlying cardiac disease, type of surgical procedure performed, exposure time on cardiac bypass, receipt of blood products, or type and duration of antimicrobials. On univariate analysis, case-patients were more likely than control-patients to have a significantly lower median age, higher median BMI, longer median duration of surgical procedure, placement of a wound VAC in the post-operative period, or to have successfully met one of the requirements of the SCIP (Table 1). On univariate analysis alone, case-patients were also statistically more likely to have had exposure to a particular surgeon (Surgeon A) and surgical fellow (Fellow X) during their surgery. All other variables analyzed were not statistically significant and are therefore not included in the table.

**Table 1.** Univariate and multivariate analyses of case-control study

	Variables (risk factors)	Cases (n = 7)	Controls (n = 21)	Univariate (p-value)	Multivariate (p-value)
**Dichotomous**	Surgeon A	7	9	<0.01	NS
	Fellow X	3	0	0.01	NS
	Wound VAC used postoperatively	3	1	0.01	NS
	Adherence to SCIP measure 10 (perioperative normothermia; 36.7 ± 0.6°C)	6	7	0.02	0.01*
**Continuous**	Lower median age (years)	56 (range: 43–67)	71 (range: 60–76)	0.0005	<0.0001*
	Longer median duration of surgery (minutes)	460 (range: 331–553)	315 (range: 198–486)	0.007	NS
	Longer median duration of cardiac bypass (minutes)	159 (range: 87–267)	123 (range: 47–247)	0.07	NS
	Higher median body mass index (kg/m2)	36 (range: 29–39)	29 (range: 16–38)	0.006	NS

* = significant in a multivariate analysis.

After controlling for confounding variables by performing multivariate analysis using logistic regression, independent risk factors for SSI among case-patients were lower age (*P* < 0.0001) and meeting the requirements of SCIP measure 10 (SCIP inf-10; perioperative temperature management to maintain normothermia [36.7 ± 0.6°C] with the exception of intentional hypothermia while on cardiac bypass) (*P* = 0.01). None of the other potential risk factors, including Surgeon A and Fellow X, were ultimately significant on multivariate analysis.

To further control for confounding, we directly compared the two surgeons who carried out the CABG procedures (Table 2). Univariate analysis revealed that patients operated on by surgeon A were more likely to have a longer duration of surgery (*P* = 0.0002), have spent a longer overall time in the operating room (*P* = 0.0002), have met SCIP measure 10 for maintaining perioperative normothermia (*P* = 0.01), have been on cardiopulmonary bypass longer (*P* = 0.02), and have HTN (*P* = 0.03) than patients operated on by surgeon B. There was a trend toward significance for younger patients as well (*P* = 0.05). Surgeon A was also more likely to have patients with a wound VAC in the post- operative period although the difference was not statistically significant. In contrast, Surgeon B was more likely to have patients with COPD (*P* = 0.01).

**Table 2.** Univariate and multivariate analyses comparing Surgeon A with Surgeon B

	Variables (risk factors)	Surgeon A (n = 16)	Surgeon B (n = 12)	Univariate (p-value)	Multivariate (p-value)
**Dichotomous**	Hypertension	16	9	0.03	NS
	COPD	2	7	0.01	NS
	Wound VAC used postoperatively	4	0	0.06	NS
	Adherence to SCIP measure 10 (perioperative normothermia; 36.7 ± 0.6°C)	11	2	0.01	NS
**Continuous**	Lower median age (years)	64 (range: 43–76)	71 (range: 66–70)	0.05	NS
	Longer median duration of surgery (minutes)	426 (range: 315–553)	315 (range: 198–486)	0.0002	0.0005*
	Longer median time spent in the operating room (minutes)	500 (range: 375–628)	359 (70–580)	0.0002	NS
	Longer median duration of cardiac bypass (minutes)	166 (range: 87–267)	91 (range: 47–247)	0.02	NS
	Higher median body mass index (kg/m2)	30 (range: 24–39)	29 (range: 16–38)	NS	NS

* = significant in a multivariate analysis.

Additional comparisons were made between the case-patients and non-case-patients for Surgeon A with regards to patient age, patient BMI, duration of bypass, and duration of surgery. Median duration of cardiac bypass and duration of surgery for case and non-case-patients in this comparison were similar. Patients who acquired SSIs were significantly more likely to be younger (*P* = 0.02) or have a higher BMI (*P* = 0.03), however (Table 3).

**Table 3.** Analysis of the 16 study patients who underwent surgery with Surgeon A

Continuous variables	Cases (n = 7)	Non-cases (n = 9)	(p-value)
Median age (years)	56 (range: 43–67)	71 (60–76)	0.02*
Median body mass index (kg/m2)	36 (range: 29–39)	29 (24–34)	0.03*
Median duration of cardiac bypass (minutes)	159 (range: 87–267)	172 (106–226)	0.9
Median duration of surgery (minutes)	460 (range: 331–553)	388 (315–473)	0.2

* = significant in analysis.

### Procedural review results

As a result of the audits conducted by institutional infection preventionists, some lapses were identified with regards to hand hygiene (alcohol-based, not surgical scrub), and equipment cleaning (specifically tubing that had touched the floor). These lapses were corrected immediately in the second month of the study period and did not recur.

With regards to OR traffic reduction in the 11^th^ month of the study period, the perfusion team immediately and permanently stopped removing equipment prior to chest closure. The interventions implemented in the 13^th^ month of the study period, which included a reduction in trainee teaching time intra-operatively as well as phasing out VAC use, also became permanent changes.

After implementation of the above measures, no additional post-CABG cases were identified and SSI rates returned to baseline. In addition, no further SSI clusters were identified in the following three years.

## Discussion

Previously identified risk factors for SSI following CABG include obesity, prolonged operative time, diabetes mellitus, hypertension, renal failure, and female gender.^2,15–17^ Study results have been variable, however, in part due to institution-specific and local influences.^2^ Optimizing infection control and antimicrobial stewardship practices can significantly improve infection rates.^18^

Our results from this retrospective case-control study show that in this institution, lower age, increased BMI, longer duration of surgery, postoperative wound VAC use, and appropriate perioperative temperature management were univariate predictors of SSI risk, while only lower age and appropriate perioperative temperature management were independent predictors of infection in patients undergoing CABG surgery upon multivariate analysis. Surgical staff were not associated with an increased relative risk of infection on multivariate analysis after adjustment for covariates was performed.

Identified risk factors from our analyses and procedural review are discussed in more detail below.

### Age

Increased age is a known risk factor for infection after cardiac surgery. In a study by Meszaros et al., for example, age greater than 74 years was the most significant risk factor for sternal infection after CABG.^17^ Functional status and comorbidities may potentially play a role in this association. Data for young adults is limited, but younger patients who develop premature coronary artery disease are more likely to have complex medical comorbidities, although overall mortality is typically less.^19–21^ In our study, the median age of patients in the control group was 71 years whereas the median age for patients who developed SSIs was 56 years. The difference between these two groups was statistically significant as a risk predictor on multivariate analysis, indicating that younger patients were in fact associated with a higher risk for an SSI. Risk factors including elevated BMI, comorbidities such as HTN and COPD, and increased complexity of surgery requiring prolonged intraoperative time may have contributed.

### Perioperative temperature management

Perioperative normothermia is a well-documented strategy to prevent complications including wound infections, coagulopathy, ischemic cardiovascular events, and mortality.^22–25^ Since hypothermia results in vasoconstriction, decreased oxygen delivery to the surgical wound site may contribute to the increased risk of infection.^23,26^ SCIP inf-10, which is part of the evidence-based SCIP measures to improve perioperative care, outlines the acceptable range of perioperative temperatures.^23^ A number of studies, however, have reported poor correlation between SCIP compliance and patient outcomes, including SSIs.^23,27–30^. In our study, six out of seven patients who developed SSIs appropriately met the SCIP inf-10 measure for perioperative normothermia, and meeting this measure was also significant on multivariate analysis as a risk factor for SSI. Our findings are therefore consistent with prior studies suggesting a potentially poor correlation between SCIP measure compliance and reduction in SSIs. Additional limiting factors could include small sample size and variability in the location of temperature monitoring.

### Body mass index

Obesity is a known risk factor for infection.^31^ Fat is more poorly vascularized, resulting in decreased tissue oxygenation and a higher risk of fat necrosis.^32,33^ Peri-operative antimicrobials are more likely to be underdosed in obese patients.^2^ In addition, obesity itself may complicate surgical procedures and increase operative time.^32^ Since higher median BMI was a statistically significant risk factor in this study on univariate analysis, although not on multivariate analysis, our findings are consistent with prior studies.

### Duration of surgery

Prolonged surgical duration was significantly associated with increased risk of SSI in our univariate analysis, although not on multivariate analysis. CABG typically requires increased operative time given multiple surgical incisions and venous grafting. Wounds that are left open are more likely to become contaminated with skin flora or other bacteria in the operating room.^15,34^ In addition, tissue desiccation at the site of the incision may increase this risk.^29,35^ Operation duration may be associated with provider fatigue.^29^ Prior studies have noted additional possible contributing factors, including intraoperative teaching.^36^ In this study, Surgeon A self-reported increased operation durations in part due to trainee involvement, as opposed to Surgeon B, who had less teaching time. This may have contributed to the longer average duration of surgery that was statistically significant on multivariate analysis when comparing Surgeon A and Surgeon B, although neither Surgeon A nor Fellow X were associated with an increased risk of SSI after CABG on multivariate analysis in the overall case-control study. Surgeon A subsequently placed additional limitations on the time allotted to trainees to decrease overall OR time.

### Wound VAC use

Use of negative pressure wound therapy after cardiac surgery has previously been shown to be safe and effective.^37,38^ Reported benefits of using a VAC system include increased blood flow and granulation tissue formation, decreased bacterial colonization, and a reduction in dressing changes.^8,38^ Complications of prolonged VAC therapy have been reported, however, and include unhealthy granulation tissue formation, nonunion, and osteomyelitis. Prolonged exposure to suction may contribute to tissue damage.^38^ Our cardiothoracic surgery team had expanded VAC use around the time of the study period based on new manufacturer data. VACs were used primarily in patients with higher BMI’s and large open wounds, which may already have increased the risk of infection. The initial association between SSI and VAC use in univariate but not multivariate analysis may therefore be related to patient characteristics and surgical complexity, but tissue damage from prolonged VAC use remains a possibility. VAC use was subsequently phased out at our institution.

### Operating room traffic

Our investigation raised concerns about operating room traffic during CABG surgeries, specifically in relation to perfusion team protocols. Studies have shown that door opening during surgery may disrupt airflow dynamics and increase colony forming units in the operating room.^39,40^ Associated distraction has also been linked to surgical mistakes.^39^ As a result, our protocols were modified to reduce prolonged door opening prior to chest closure.

Limitations of this study include the relatively small number of cases in the cluster, which may have affected precise estimates of key predictor effects for important categorical predictors in the multivariate model, rendering model parameter estimations unstable. We found, however, that statistically significant differences ascertained on univariate analyses enabled generation of a plausible hypothesis to explain the underlying risk factors for infection. In addition, only male patients were present in the cluster and so gender could not be assessed as a risk factor. Finally, because this was a retrospective case-control study, we were not able to document long-term sequelae of infection in case-patients.

In conclusion, multiple risk factors can affect SSIs after CABG, although prior studies evaluating these factors have yielded varying results. While investigating a cluster of SSIs at our institution, we found multiple variables potentially associated with increased SSI risk on univariate analysis, and after performing multivariate analysis using logistic regression, we identified younger age and meeting SCIP measure 10 for maintenance of perioperative normothermia as statistically significant independent risk factors for SSI. After the implementation of measures directed toward minimizing operating room traffic and phasing out wound VAC use, no additional clusters of SSIs were observed.

## References

[1] D’Agostino RS , Jacobs JP , Badhwar V , et al. The society of thoracic surgeons adult cardiac surgery database: 2018 update on outcomes and quality. Ann Thorac Surg 2018;105:15–23. 29233331 10.1016/j.athoracsur.2017.10.035

[2] Ahmed D , Cheema FH , Ahmed YI , et al. Incidence and predictors of infection in patients undergoing primary isolated coronary artery bypass grafting: a report from a tertiary care hospital in a developing country. 2011;52:99–104. 21224817

[3] Gelijns AC , Moskowitz AJ , Acker MA , et al. Management practices and major infections after cardiac surgery. J Am Coll Cardiol 2014;64:372–381. 25060372 10.1016/j.jacc.2014.04.052 PMC4222509

[4] Greco G , Shi W , Michler RE , et al. Costs associated with health care–associated infections in cardiac surgery. J Am Coll Cardiol 2015;65:15–23. 25572505 10.1016/j.jacc.2014.09.079 PMC4293042

[5] De Lissovoy G , Fraeman K , Hutchins V , Murphy D , Song D , Vaughn BB . Surgical site infection: incidence and impact on hospital utilization and treatment costs. Am J Infect Control 2009;37:387–397. 19398246 10.1016/j.ajic.2008.12.010

[6] Klevens RM , Edwards JR , Richards CL , et al. Estimating health care-associated infections and deaths in U.S. Hospitals, 2002. Public Health Rep 2007;122:160–166. 17357358 10.1177/003335490712200205 PMC1820440

[7] Gummert JF , Barten MJ , Hans C , et al. Mediastinitis and cardiac surgery - an updated risk factor analysis in 10,373 consecutive adult patients. Thorac Cardiovasc Surg 2002;50:87–91. 11981708 10.1055/s-2002-26691

[8] Sjögren J , Malmsjö M , Gustafsson R , Ingemansson R . Poststernotomy mediastinitis: a review of conventional surgical treatments, vacuum-assisted closure therapy and presentation of the Lund University Hospital mediastinitis algorithm. Eur J Cardiothorac Surg 2006;30:898–905. 17056269 10.1016/j.ejcts.2006.09.020

[9] Braxton JH , Marrin CAS , McGrath PD , et al. 10-Year follow-up of patients with and without mediastinitis. Semin Thorac Cardiovasc Surg 2004;16:70–76. 15366690 10.1053/j.semtcvs.2004.01.006

[10] Zacharias A , Habib RH . Factors predisposing to median sternotomy complications. Chest 1996;110:1173–1178. 8915216 10.1378/chest.110.5.1173

[11] Nagachinta T , Stephens M , Reitz B , Polk BF . Risk factors for surgical-wound infection following cardiac surgery. J Infect Dis 1987;156:967–973. 3680996 10.1093/infdis/156.6.967

[12] Slaughter MS , Olson MM , Lee JT , Ward HB . A fifteen-year wound surveillance study after coronary artery bypass. Ann Thorac Surg 1993;56:1063–1068. 8239800 10.1016/0003-4975(95)90014-4

[13] Horan TC , Andrus M , Dudeck MA . CDC/NHSN surveillance definition of health care–associated infection and criteria for specific types of infections in the acute care setting. Am J Infect Control 2008;36:309–332. 18538699 10.1016/j.ajic.2008.03.002

[14] NHSN Surgical Site Infection Event (SSI). National Healthcare Safety Network, CDC website. https://www.cdc.gov/nhsn/pdfs/pscmanual/9pscssicurrent.pdf. Published 2022. Accessed April 12, 2024.

[15] Pan L , Tan S , Cao L , Feng X . Risk factor analysis and management strategies of operating room-related infections after coronary artery bypass grafting. J Thorac Dis 2018;10:4949–4956. 30233869 10.21037/jtd.2018.08.01 PMC6129937

[16] Salehi Omran A , Karimi A , Ahmadi SH , et al. Superficial and deep sternal wound infection after more than 9000 coronary artery bypass graft (CABG): incidence, risk factors and mortality. BMC Infect Dis 2007;7:112. 17888179 10.1186/1471-2334-7-112 PMC2075514

[17] Meszaros K , Fuehrer U , Grogg S , et al. Risk factors for sternal wound infection after open heart operations vary according to type of operation. Ann Thorac Surg 2016;101:1418–1425. 26652136 10.1016/j.athoracsur.2015.09.010

[18] Frenette C , Sperlea D , Tesolin J , Patterson C , Thirion DJG . Influence of a 5-year serial infection control and antibiotic stewardship intervention on cardiac surgical site infections. Am J Infect Control 2016;44:977–982. 27125912 10.1016/j.ajic.2016.02.029

[19] Dani SS , Minhas AMK , Arshad A , et al. Trends in characteristics and outcomes of hospitalized young patients undergoing coronary artery bypass grafting in the United States, 2004 to 2018. J Am Heart Assoc 2021;10:e021361. 34459230 10.1161/JAHA.121.021361 PMC8649273

[20] Dalén M , Ivert T , Holzmann MJ , Sartipy U . Coronary artery bypass grafting in patients 50 years or younger: a swedish nationwide cohort study. Circulation 2015;131:1748–1754. 25788458 10.1161/CIRCULATIONAHA.114.014335

[21] Biancari F , Gudbjartsson T , Heikkinen J , et al. Comparison of 30-day and 5-year outcomes of percutaneous coronary intervention versus coronary artery bypass grafting in patients aged ≤50 years (the coronary aRtery diseAse in younG adultS study). Am J Cardiol 2014;114:198–205. 24878127 10.1016/j.amjcard.2014.04.025

[22] Sessler DI . Perioperative temperature monitoring. Anesthesiology 2021;134:111–118. 32773677 10.1097/ALN.0000000000003481

[23] Scott AV , Stonemetz JL , Wasey JO , et al. Compliance with surgical care improvement project for body temperature management (SCIP Inf-10) is associated with improved clinical outcomes. Anesthesiology 2015;123:116–125. 25909970 10.1097/ALN.0000000000000681

[24] Kurz A . Perioperative normothermia to reduce the incidence of surgical-wound infection and shorten hospitalization. N Engl J Med 1996;334:1209–1215. 8606715 10.1056/NEJM199605093341901

[25] Frank SM , Fleisher LA , Breslow MJ , Olson KF , Beattie C . Perioperative Maintenance of Normothermia Reduces the Incidence of Morbid Cardiac Events. 1997;277:1127–1134. 9087467

[26] Sheffield CW , Sessler DI , Hopf HW , et al. Centrally and locally mediated thermoregulatory responses alter subcutaneous oxygen tension. Wound Repair Regen 1996;4:339–345. 17177730 10.1046/j.1524-475X.1996.40310.x

[27] Rasouli MR , Jaberi MM , Hozack WJ , Parvizi J , Rothman RH . Surgical care improvement project (SCIP): Has its mission succeeded? J Arthroplasty 2013;28:1072–1075. 23602416 10.1016/j.arth.2013.03.004

[28] Tillman M , Wehbe-Janek H , Hodges B , Smythe WR , Papaconstantinou HT . Surgical care improvement project and surgical site infections: can integration in the surgical safety checklist improve quality performance and clinical outcomes? J Surg Res 2013;184:150–156. 23582762 10.1016/j.jss.2013.03.048

[29] Nguyen DB , Shugart A , Lines C , et al. National healthcare safety network (NHSN) dialysis event surveillance report for 2014. Clin J Am Soc Nephrol 2017;12:1139–1146. 28663227 10.2215/CJN.11411116 PMC5498356

[30] Brown MJ , Curry TB , Hyder JA , et al. Intraoperative hypothermia and surgical site infections in patients with Class I/clean wounds: a case-control study. J Am Coll Surg 2017;224:160–171. 27825917 10.1016/j.jamcollsurg.2016.10.050

[31] Chan PG , Sultan I , Gleason TG , et al. Contemporary outcomes of coronary artery bypass grafting in obese patients. J Card Surg 2020;35:549–556. 31945232 10.1111/jocs.14415

[32] Thelwall S , Harrington P , Sheridan E , Lamagni T . Impact of obesity on the risk of wound infection following surgery: results from a nationwide prospective multicentre cohort study in England. Clin Microbiol Infect 2015;21:1008.e1–8. 10.1016/j.cmi.2015.07.003 26197212

[33] Kabon B , Nagele A , Reddy D , et al. Obesity Decreases Perioperative Tissue Oxygenation. 2004;100:274–280. 10.1097/00000542-200402000-00015 PMC1395476 14739800

[34] Cheng H , Chen BPH , Soleas IM , Ferko NC , Cameron CG , Hinoul P . Prolonged operative duration increases risk of surgical site infections: a systematic review. Surg Infect 2017;18:722–735. 10.1089/sur.2017.089 PMC5685201 28832271

[35] Haridas M , Malangoni MA . Predictive factors for surgical site infection in general surgery. Surgery 2008;144:496–503. 18847631 10.1016/j.surg.2008.06.001

[36] Campbell DA , Henderson WG , Englesbe MJ , et al. Surgical site infection prevention: the importance of operative duration and blood transfusion—results of the first american college of surgeons–national surgical quality improvement program best practices initiative. J Am Coll Surg 2008;207:810–820. 19183526 10.1016/j.jamcollsurg.2008.08.018

[37] Deniz H , Gokaslan G , Arslanoglu Y , et al. Treatment outcomes of postoperative mediastinitis in cardiac surgery; negative pressure wound therapy versus conventional treatment. J Cardiothorac Surg 2012;7:67. 22784512 10.1186/1749-8090-7-67 PMC3432617

[38] Bapat V , El-Muttardi N , Young C , Venn G , Roxburgh J . Experience with vacuum-assisted closure of sternal wound infections following cardiac surgery and evaluation of chronic complications associated with its use. J Card Surg 2008;23:227–233. 18435637 10.1111/j.1540-8191.2008.00595.x

[39] Young RS , O’Regan DJ . Cardiac surgical theatre traffic: time for traffic calming measures? Interact Cardiovasc Thorac Surg 2010;10:526–529. 20100706 10.1510/icvts.2009.227116

[40] Scaltriti S , Cencetti S , Rovesti S , Marchesi I , Bargellini A , Borella P . Risk factors for particulate and microbial contamination of air in operating theatres. J Hosp Infect 2007;66:320–326. 17655973 10.1016/j.jhin.2007.05.019

